# Inzidenz, Schweregrad und möglicher Pathomechanismus von Immuntherapie‐assoziiertem Pruritus bei Melanompatienten: Incidence, severity and potential pathomechanism of immune‐related pruritus in melanoma patients undergoing immune checkpoint inhibitor therapy

**DOI:** 10.1111/ddg.70048x

**Published:** 2026-11-10

**Authors:** Andrea Roggo, Nikolina Ivanovic, Patrick Turko, Lukas Kraehenbuehl, Mitchell Levesque, Reinhard Dummer, Barbara Meier‐Schiesser

**Affiliations:** ^1^ Departement für Dermatologie Universitätsspital Zürich Universität Zürich, Schweiz; ^2^ Departement für Dermatologie Kantonsspital Aarau Aarau Schweiz

**Keywords:** Eosinophile Granulozyten, Kombinationstherapie, topische Steroide, immune checkpoint inhibitors, immune‐related pruritus, melanoma

## Abstract

**Hintergrund und Ziele:**

Pruritus ist eine häufige immunvermittelte unerwünschte Wirkung (irAE) unter Therapie mit Checkpoint‐Inhibitoren (ICI) und beeinträchtigt die Lebensqualität der Patienten erheblich. In dieser Studie untersuchen wir die Häufigkeit, den Schweregrad und Therapieansätze von Pruritus bei Melanompatienten unter ICI‐Therapie.

**Patienten und Methodik:**

Klinische Daten von 461 Melanompatienten am Universitätsspital Zürich wurden im Rahmen einer retrospektiven, monozentrischen Studie analysiert. Die Patienten erhielten ICI entweder als Kombinationstherapie (Ipilimumab und Nivolumab) oder Monotherapie (Nivolumab oder Pembrolizumab).

**Ergebnisse:**

Immunvermittelter Pruritus (irPruritus) war mit einer verlängerten Lebenserwartung assoziiert. irPruritus trat bei 23,0 % der Patienten auf (106 von 461) und wurde unter Kombinationstherapie signifikant häufiger und früher beobachtet als unter Monotherapie (p < 0,001). Zudem zeigte sich ein deutlicher Anstieg der Eosinophilen von Therapiebeginn bis zum Beginn des irPruritus. In 90,9 % der betroffenen Patienten erwiesen sich topische Glukokortikoide als wirksame Therapie. Darüber hinaus bestand ein signifikanter Zusammenhang zwischen dem Auftreten von irPruritus und immunvermittelter Fatigue (irFatigue; p < 0,01) als auch immunvermitteltem Exanthem (irRash; p < 0,001).

**Schlussfolgerungen:**

Pruritus zählt zu den häufigsten Nebenwirkungen ausgelöst durch ICI. Gleichzeitiges Auftreten von irFatigue und irRash weist auf einen umfassenderen systemischen Effekt hin. Die Lebensqualität der Betroffenen kann stark beeinträchtigt sein. Prospektive Studien sind erforderlich, um die Ursachen sowie Rolle von Eosinophilen zu klären und wirksame Behandlungsstrategien mit erhaltenen Therapiewirksamkeit zu entwickeln.

## Summary

**Background and objectives:**

Pruritus is a frequent cutaneous immune‐related adverse event (irAE) associated with immune checkpoint inhibitors (ICI), impacting patients' quality of life. This study investigates the incidence, severity and management of pruritus in melanoma patients treated with ICI.

**Patients and methods:**

Clinical data from 461 melanoma patients at the University Hospital of Zurich were analyzed in a retrospective, monocentric study. Patients received ICI either as combination therapy (ipilimumab and nivolumab) or monotherapy (nivolumab or pembrolizumab).

**Results:**

Immune‐related pruritus (IrPruritus) was associated with increased overall survival. It affected 23.0 % of patients (106/461), with a significantly higher frequency and earlier onset observed in combination therapy compared to monotherapy (p < 0.001). A significant, pronounced increase in eosinophil counts was noted from baseline to onset of irPruritus. Topical steroids proved effective in 90.9 % of cases. Additionally, irPruritus was significantly correlated with both irFatigue (p < 0.01) and irRash (p < 0.001).

**Conclusions:**

Pruritus is a common side effect of ICI therapy. Its correlation with irFatigue and irRash highlights the broader systemic impact impairing patients’ quality of life. Further prospective studies are needed to elucidate the underlying mechanisms such as involvement of eosinophils and identify optimal treatment strategies while maintaining ICI effectiveness.

## EINLEITUNG

Das Melanom gehört weltweit zu den häufigsten malignen Tumorerkrankungen mit hoher Mortalitätsrate und ist eine erhebliche globale Gesundheitsbelastung.^1^ In den vergangenen zwei Jahrzehnten haben Checkpoint‐Inhibitoren (ICI) die Lebenserwartung bei metastasiertem Melanom signifikant verbessert.^2^ Insbesondere die Zulassung von Pembrolizumab und Nivolumab (PD‐1‐Antikörper) sowie Ipilimumab (CTLA‐4‐Antikörper) als Mono‐ oder Kombinationstherapie haben die Lebenserwartung signifikant verbessert.^2^ *Programmed Cell Death Protein 1* (PD‐1) und *Cytotoxic T‐Lymphocyte Antigen 4* (CTLA‐4) sind Rezeptoren auf der Oberfläche von zytotoxischen T‐Zellen, die die Immunantwort hemmen.^3^ Sie spielen eine entscheidende Rolle zur Aufrechterhaltung der körpereigenen Selbsttoleranz und Kontrolle von Entzündungen in gesundem Gewebe.^3^

CTLA‐4 wirkt hauptsächlich in der frühen Phase der T‐Zell‐Aktivierung. Es reduziert die Mobilisierung von naiven T‐Zellen und Gedächtnis‐T‐Zellen und verhindert so eine Überaktivierung des Immunsystems. Die spezifische Hemmung von CTLA‐4 führt zu einer breiteren Immunantwort mit größerer Vielfalt von T‐Zell Rezeptoren und vermehrter Tumorinfiltration.^4^ Ergänzend beeinflusst PD‐1 die Immunantwort im peripheren Gewebe und verhindert Autoimmunreaktionen. PD‐1 Inhibierung erhöht die Anzahl T‐Zellen im Blut ohne Einfluss auf die Diversität der T‐Zell Rezeptoren im restlichen Körper.^5^ Beide, PD‐1 und CTLA‐4, unterdrücken die T‐Zell‐Aktivität bei Entzündungen und im Tumorgewebe und helfen so dem Tumor die Immunabwehr zu umgehen.^6^ Gleichzeitige Inhibierung von PD‐1 und CTLA‐4 beim metastasiertem Melanom hat einen synergistischen Effekt und wird als Kombinationstherapie mit Nivolumab und Ipilimumab angewendet.^7^

Die Immuntherapie ist hochwirksam, geht jedoch häufig mit immunvermittelten Nebenwirkungen (immune‐related adverse events; irAE) einher. Betroffene Organe sind unter anderem die Haut, der Gastrointestinaltrakt, Hormondrüsen oder die Lunge.^8^, ^9^ Das Auftreten irAE ist mit einer verbesserten Lebenserwartung assoziiert. Unter Behandlung mit PD‐1‐Inhibitoren treten leichte bis mäßige irAE auf. Schwerwiegende irAE (Grad 3 oder 4 gemäß *Common Terminology Criteria for Adverse Events* [CTCAE]) werden nur bei etwa 23 % der Behandelten beobachtet.^9^ Im Vergleich dazu kommt es unter Kombinationstherapie mit Ipilimumab und Nivolumab in 59 % der Fälle zu schwerwiegenden irAE.^9^ Frühe Erkennung und spezifische Therapie der Nebenwirkungen sind wichtig, um dauerhafte Schäden und eine Verschlechterung der Lebensqualität zu verhindern. Für die meisten irAE empfehlen die Leitlinien eine Behandlung mit Kortikosteroiden oder Immunsuppressiva.^11^, ^12^, ^13^ Die Therapie mit ICI sollte bei irAE ≥ Grad 3 unterbrochen werden.^14^, ^15^

Dermatologische irAE betreffen mehr als einen Drittel der ICI‐Patienten und sind die häufigsten irAE.^16^ Am häufigsten sind Exantheme (irRash) und Pruritus (irPruritus).^16^ Weitere typische kutane irAE sind lichenoide Reaktionen, makulopapulöse Exantheme, ekzematöse Dermatitiden sowie Vitiligo‐ähnliche Depigmentierungen, insbesondere beim metastasiertem Melanom.^17^ Immunvermittelter Pruritus tritt bei 11 %–47 % der mit ICI behandelten Patienten auf.^16^, ^18^, ^19^ Juckreiz erhöht die Morbidität der Patienten, beeinflusst die Lebensqualität und hat so einen negative Einfluss auf den Therapieverlauf.^16^, ^18^, ^19^ Trotz der klinischen Relevanz von irPruritus unter ICI ist wenig bekannt über die klinischen Merkmale.^18^, ^19^ Spezifische Behandlungsempfehlungen beinhalten Basismaßnahmen wie Emollenzien.^20^ Zusätzlich können topische Steroide (TCS) oder Antihistaminika eingesetzt werden. In schweren Fällen ersetzen GABA‐Analoge die Antihistamine.^20^ Einzelfälle über alternative systemische Therapieansätze wie Dupilumab,^21^ Omalizumab,^22^ oder NK1‐Antagonisten sind beschrieben.^23^ Als alternative Therapieoption kommt eine Phototherapie, insbesondere eine Schmalspektrum‐UV‐B‐Therapie (nbUVB), infrage.^24^ Der genaue Pathomechanismus des irPruritus ist bislang nicht vollständig geklärt, und eine spezifisch zugelassene Therapie steht derzeit nicht zur Verfügung.

Diese retrospektive Studie untersucht systematisch den Zusammenhang zwischen ICI und irPruritus bei Patienten mit metastasiertem Melanom. Zur besseren Charakterisierung des immunvermittelten Pruritus analysieren wir den klinischen Verlauf, potenzielle prädisponierende Faktoren sowie die Korrelation mit dem Therapieansprechen. Die gewonnenen Erkenntnisse sollen eine evidenzbasierte Grundlage für ein optimiertes Management des immunvermittelten Pruritus schaffen, ohne die Wirksamkeit der ICI‐Therapie zu beeinträchtigen.^25^

## PATIENTEN UND METHODIK

Diese retrospektive Kohortenstudie untersuchte Patienten mit metastasiertem Melanom, die mit Pembrolizumab, Nivolumab oder einer Kombination aus Nivolumab und Ipilimumab an der Klinik für Dermatologie des Universitätsspitals Zürich (USZ), einem tertiären akademischen Krebszentrum, behandelt wurden. Klinische Daten von Patienten mit metastasiertem Melanom zwischen 2014 und 2022 wurden analysiert. Patienten mit passendem Therapieschema wurden in der EUMELAREG‐Datenbank identifiziert. Klinische Daten wurden aus den medizinischen Unterlagen im KISIM‐Patientendatensystem extrahiert.

Einschlusskriterien waren eine bestätigte Melanomdiagnose, dokumentierte Therapie mit Pembrolizumab, Nivolumab oder Ipilimumab, Alter über 18 Jahre und Verfügbarkeit der klinischen Dokumentation. Patienten, die den Generalkonsent des Universitätsspitals Zürich nicht erteilt haben beziehungsweise ihn ausdrücklich abgelehnt haben, wurden nicht in die Studie eingeschlossen.

Erfasst wurden Patientencharakteristika (Alter, Geschlecht, Todesdatum [Stand 10. Mai 2025], medizinische Vorgeschichte einschließlich pruritischer Vorerkrankungen), Tumorcharakteristika (TNM‐Stadium, Lokalisation, Mutationsstatus), Therapieparameter des metastasierten Melanoms (Therapiebeginn, Therapiedauer, Anzahl der Infusionen) sowie Charakteristika von irAE und Pruritus (Beginn, Lokalisation, Schweregrad, Therapie). Die Bestimmung der Eosinophilenkonzentration erfolgte vor Therapiebeginn sowie zum Zeitpunkt des Auftretens von irPruritus. Bei Patienten ohne irPruritus wurden die Werte an Infusionszeitpunkten erhoben, die dem medianen Auftreten von irPruritus in der jeweiligen Behandlungsgruppe entsprachen (Ipilimumab/Nivolumab: Infusion 2, Nivolumab: Infusion 7, Pembrolizumab: Infusion 10). Die Klassifikation des Schweregrads von irPruritus und irRash erfolgte gemäß den CTCAE. Topische Kortikosteroide wurden entsprechend ihrer Wirkstärke in schwach, mittelstark, stark und sehr stark eingeteilt. Eine Zweitlinientherapie wurde nur dann berücksichtigt, wenn zwischen den jeweiligen Behandlungssequenzen ein Intervall von mindestens 6 Monaten lag. Die Datenerfassung erfolgte in Microsoft Excel (Microsoft Corporation, Seattle, WA, USA).

Wir führten alle statistischen Auswertungen mit R (Version 4.3.1) durch. Für numerische Variablen wurde der Welch‐t‐Test verwendet, für kategoriale Variablen der Chi‐Quadrat‐Test auf Unabhängigkeit. Time‐to‐Event‐Daten wurden mittels multivariater Cox‐Proportional‐Hazards‐Regression analysiert. Die relative Eosinophilenkonzentration bei Patienten mit und ohne irPruritus wurde mithilfe eines generalisierten linearen gemischten Modells (GLMM; glmmTMB‐Paket) untersucht. Das Modell umfasste den Zeitpunkt (Baseline vs Follow‐up), den irPruritus‐Status (ja/nein) sowie deren Interaktion als feste Effekte und berücksichtigte wiederholte Messungen pro Patient als zufälligen Effekt. p‐Werte ≤ 0,05 wurden als statistisch signifikant definiert.

### Ethikvotum und Einwilligungserklärung

Das Projekt wurde von der Ethikkommission des Kantons Zürich geprüft und genehmigt. Alle in diesem Manuskript beschriebenen Patienten erteilten ihr schriftliches Einverständnis zur Veröffentlichung ihrer Falldetails.

## ERGEBNISSE

### Kombinationstherapie verursacht häufiger irPruritus als Monotherapie

Insgesamt 461 Patienten wurden in die Studie eingeschlossen (Tabelle 1). Das Durchschnittsalter war 62,2 Jahre (Standardabweichung [SD] 15,0), mit 284 (61,6 %) männlichen und 177 (38,4 %) weiblichen Patienten. Unter den Patienten mit irPruritus waren 41 (38,7 %) weiblich und 65 (61,6 %) männlich. Das durchschnittliche Alter betrug 63 Jahre (SD 15,2). In der gesamten Kohorte waren 58,3 % der Patienten mit metastasiertem Melanom im Stadium IV. Ein deutlicher Anteil hatte eine *NRAS*‐ (21,7 %) oder *BRAF*‐Mutation (34,3 %). Wir beobachteten keinen Zusammenhang zwischen dem Auftreten von irPruritus und den klinischen Parametern Alter, Geschlecht, Tumorstadium, Mutationsstatus und medizinische Vorgeschichte (p > 0,05). Das primäre Melanom war am häufigsten an den Extremitäten (37,8 %), gefolgt vom Stamm (27,3 %), Kopf‐Hals‐Bereich (21,5 %) und in den Schleimhäuten (2,2 %). Es zeigte sich keine Abhängigkeit zwischen der Primärlokalisation und irPruritus (p = 0.49). Ebenfalls keine signifikante Korrelation bestand zwischen irPruritus und vorausgegangenen juckenden Erkrankungen, nichtmelanozytären malignen Tumoren, Niereninsuffizienz, Diabetes oder neurologischen Erkrankungen. Insgesamt erhielten 199 Patienten (43,3 %) Pembrolizumab, 148 Patienten (32,1 %) Nivolumab und 114 Patienten (24,7 %) eine Kombinationstherapie mit Ipilimumab und Nivolumab. Die mittlere Dauer der ICI‐Therapie betrug 35,2 Wochen (SD 36,3) mit durchschnittlich 12,0 Infusionen pro Patient (SD 11,0). Zur Beurteilung der prognostischen Relevanz von irAE analysierten wir deren Zusammenhang mit der Lebenserwartung. Das Auftreten von irPruritus war mit einem nicht‐signifikanten Gesamtüberleben assoziiert (HR 0,82, p = 0,35, multivariate Cox‐Regression) (Abbildung 1a). Unter den dokumentierten irAE zeigten irEdema, irHepatitis, irMyasthenie, irMyositis, irPneumonie und irKolitis/Gastritis einen Zusammenhang mit einem reduzierten Gesamtüberleben (Abbildung S1a–e im Online‐Supplement). Im Gegensatz dazu waren irRash, irPankreatitis, irArthritis, irFatigue, irHypothyreose, irVitiligo, irNephritis, irXerostomie, irKeratitis sicca und irKeratose mit einem verlängerten Gesamtüberleben assoziiert (Abbildung S1f–o im Online‐Supplement). Immunvermittelter Pruritus trat bei 106 Patienten auf und war unter Kombinationstherapie signifikant häufiger (31,6 %) als unter Monotherapie mit Nivolumab (17,6 %) oder Pembrolizumab (22,1 %; p = 0,03) (Tabelle 2). Auffällig war der signifikant frühere Beginn unter Kombinationstherapie (nach 1,6 Infusionen [SD 0,8]) verglichen mit Pembrolizumab (nach 7,4 Infusionen [SD 7,1]) und Nivolumab (nach 8,8 Infusionen [SD 5,2]) (p < 0,001). Zwischen den drei Behandlungsregimen zeigte sich kein signifikanter Unterschied hinsichtlich der Charakteristika des irPruritus.

**TABELLE 1:** Patientencharakteristika der Gesamtkohorte sowie stratifiziert nach irPruritus‐Status. Die statistische Auswertung erfolgte mittels Welch‐t‐Test (numerische Variablen) und Chi‐Quadrat‐Test auf Unabhängigkeit (kategoriale Variablen).

	Alle Patienten (n = 461)	Patienten ohne Pruritus (n = 355)	Patienten mit Pruritus (n = 106)	p‐Wert
***Geschlecht, n (%)***				0,94
männlich	284 (61,6)	219 (61,3)	65 (61,6)	
weiblich	177 (38,4)	136 (38,3)	41 (38,7)	
***Alter, Durchschnitt in Jahren (SD)***	63 (15,0)	62 (15,6)	64 (15,2)	0,42
***Medizinische Vorgeschichte, n (%)***				
Malignom	147 (31,9)	116 (32,7)	31 (29,2)	0,51
Prurigo	129 (28,0)	97 (27,3)	32 (30,2)	0,56
Neuropathie	76 (16,5)	56 (15,8)	20 (18,9)	0,45
Niereninsuffizienz	54 (11,7)	38 (10,7)	16 (15,1)	0,22
Diabetes	53 (11,5)	37 (10,4)	16 (15,1)	0,18
***Staging, n (%)***				0,68
II	16 (3,4)	11 (3,2)	5 (4,6)	
III	176 (38,3)	136 (38,3)	40 (37,6)	
IV	269 (58,3)	208 (58,6)	61 (57,5)	
***Mutation, n (%)***				
NRAS	100 (21,7)	78 (22,0)	22 (20,8)	0,79
BRAF	158 (34,3)	130 (36,6)	28 (26,4)	0,05
***Lokalisation, n (%)***				0,49
Extremitäten	174 (37,7)	139 (39,2)	35 (33,0)	
Stamm	126 (27,3)	91 (25,6)	35 (33,0)	
Kopf/Hals	99 (21,5)	75 (21,1)	24 (22,6)	
Mucosa	9 (2,0)	8 (2,3)	2 (1,9)	
Unbekannter Primarius	53 (11,5)	42 (11,8)	11 (10,4)	
***Therapie, n (%)***				0,03
Pembrolizumab	199 (43,3)	155 (43,7)	44 (41,5)	
Nivolumab	148 (32,1)	122 (34,4)	26 (24,5)	
Ipilimumab/Nivolumab	114 (24,7)	78 (22,0)	36 (34,0)	
***Therapiewochen, Durchschnitt (SD)***	35 (36,3)	34 (36,5)	40 (35,9)	0,19
***Infusionen, n (SD)***	12 (11,0)	12 (11,2)	13 (10,6)	0,28

Abk.: SD, Standardabweichung

**ABBILDUNG 1:**
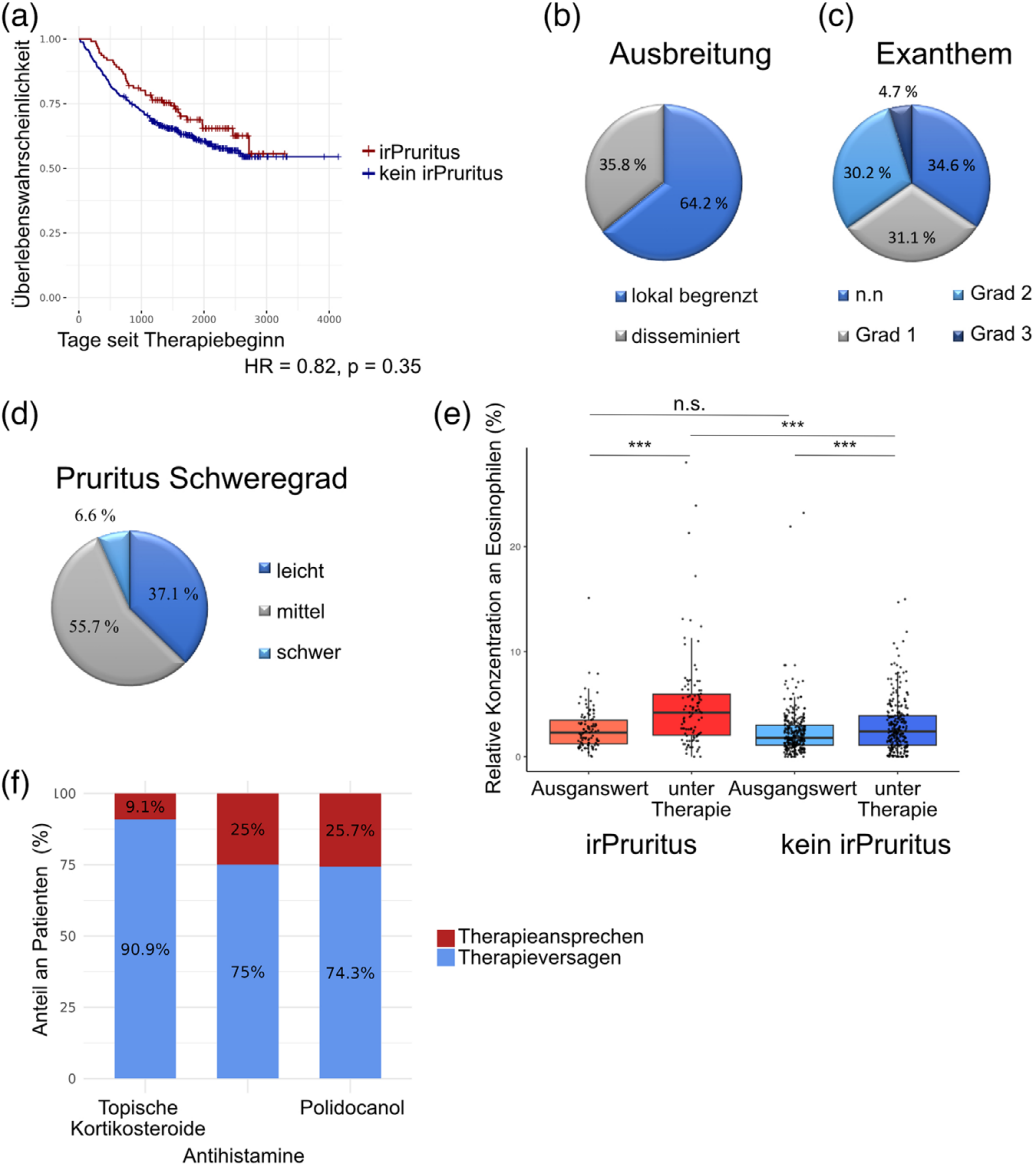
Klinische Charakteristika des irPruritus und Assoziation mit Eosinophilenzahlen. (a) Kaplan‐Meier‐Kurve zum Gesamtüberleben von Patienten mit (n = 106) und ohne irPruritus (n = 355). Die statistische Auswertung erfolgte mittels multivariater Cox‐Regression (Proportional‐Hazards‐Modell) unter Einschluss aller erhobenen immunvermittelten Nebenwirkungen (irAE) als Kovariablen. (b) Ausbreitung des irPruritus. (c) Grad des irRash. (d) Schweregrad des Pruritus. (e) Boxplots der relativen Eosinophilenzahlen. Die Werte wurden vor Therapiebeginn (Baseline), zum Zeitpunkt des Auftretens des irPruritus (Follow‐up, irPruritus‐Kohorte) oder zu Infusionszeitpunkten erhoben, die dem medianen Auftreten des irPruritus in der jeweiligen Therapiegruppe entsprachen (Follow‐up, Kohorte ohne irPruritus; Tabelle 2). Die statistische Auswertung erfolgte mittels generalisiertem linearem gemischten Modell. (f) Balkendiagramm mit der Anzahl erfolgreicher Behandlungen pro Therapie im Verhältnis zur Anzahl der behandelten Fälle. Absolute Zahlen sowie relative Prozentsätze sind angegeben. Abk.: irAE, immunvermittelte Nebenwirkungen; irPruritus, immunvermittelter Pruritus; n.n., nicht nachgewiesen; SD, Standardabweichung

**TABELLE 2:** Charakteristika des irPruritus in Abhängigkeit vom Therapieschema. Die statistische Auswertung erfolgte mittels Welch‐t‐Test (numerische Variablen), Chi‐Quadrat‐Test auf Unabhängigkeit (kategoriale Variablen) sowie Cox‐Regression (zeitabhängige Variablen).

	Ipilimumab/Nivolumab (n = 36)	Pembrolizumab (n = 44)	Nivolumab (n = 26)	p‐Wert
***Pruritus Prävalenz, n (%)***	36 (31,6)	44 (22,1)	26 (17,6)	0,03
***Pruritusbeginn, mediane Infusion (SD)***	1,0 (0,8)	9 (7,1)	6 (5,2)	< 0,001
***Pruritus‐Charakteristika, Durchschnitt (SD)***				
Exanthem	0,7 (0,5)	0,6 (0,5)	0,7 (0,5)	0,4
Grad	1,1 (0,9)	0,8 (0,8)	1,0 (0,9)	0,3
Intensität	1,9 (0,5)	1,6 (0,6)	1,6 (0,6)	0,1

Die weiterführende Analyse des irPruritus (n = 106) ergab eine lokalisierte Verteilung in 64,2 % (n = 68) sowie ein disseminiertes Muster in 35,8 % (n = 38) (Abbildung 1b). Zeitgleich berichteten 70 (66,0 %) Patienten irRash. Innerhalb aller Patienten mit irPruritus war die Intensität des irRash unterschiedlich ausgeprägt und reichte von Grad 1 (n = 33, 31,1 %) über Grad 2 (n = 32, 30,2 %) bis zu Grad 3 (n = 5, 4,7 %) (Abbildung 1c). Bei zwei Patienten war der irRash nicht klassifiziert. Zur besseren Einschätzung wurde der irPruritus entsprechend den CTCAE‐Graden in mild, moderat oder schwer klassifiziert.^9^ Von allen Patienten mit irPruritus entwickelten 59 (55,7 %) moderaten, 40 (37,1 %) milden und sieben (6,6 %) schweren Juckreiz (Abbildung 1d). Bei einem Patienten wurde die Therapie aufgrund des starken irPruritus abgebrochen.

### Eosinophile sind während irPruritus erhöht

Zur Untersuchung von Unterschieden in Laborparametern zwischen Patienten mit und ohne irPruritus wurden Blutwerte analysiert. Für Patienten ohne irPruritus wurde der Untersuchungszeitpunkt an den medianen Zeitpunkt des Auftretens von irPruritus innerhalb des jeweiligen Therapieregimes angepasst (Tabelle 2). Vor Beginn der Therapie bestand kein signifikanter Unterschied der Eosinophilenspiegel zwischen Patienten, die später einen irPruritus entwickelten, und Patienten ohne irPruritus (p = 0,4; generalisiertes lineares gemischtes Modell) (Abbildung 1e). Während der Therapie zeigten beide Gruppen einen signifikanten Anstieg der Eosinophilen (jeweils p < 0,001; generalisiertes lineares gemischtes Modell) (Abbildung 1e). Der Anstieg war jedoch bei Patienten mit irPruritus stärker ausgeprägt. In dieser Kohorte stiegen die Eosinophilenspiegel von 2,3 % zu Therapiebeginn auf 4,2 % zum Zeitpunkt des Auftretens des irPruritus.

### irPruritus spricht auf topische Steroide an

In unserer Kohorte umfasste die spezifische Therapie des irPruritus topische Kortikosteroide (n = 55), Antihistaminika (n = 20) sowie die topische Anwendung von Polidocanol (n = 70) (Abbildung 1f). Topische Kortikosteroide erwiesen sich bei 90,9 % der Patienten als wirksam, gefolgt von Antihistaminika (75,0 %) und Polidocanol (74,3 %).

Die eingesetzten topischen Kortikosteroide wurden entsprechend ihrer Wirkstärke in mittelstark (Klasse II: Clobetasonbutyrat [0,05 %], Triamcinolonacetonid [0,1–0,5 %]), stark (Klasse III: Mometasonfuroat [0,1 %], Betamethasonvalerat [0,1 %], Betamethasondipropionat [0,05 %]) und sehr stark (Klasse IV: Clobetasolpropionat [0,05 %]) eingeteilt. Eine Wirksamkeit zeigte sich in Klasse III bei 38/41 Patienten (93,2 %), in Klasse IV bei 9/10 Patienten (90,0 %) und in Klasse II bei 3/4 Patienten (75,0 %) (Table S1 im Online‐Supplement).

### irPruritus ist mit chronischer irFatigue assoziiert und tritt häufig gemeinsam mit irRash auf

Checkpoint‐Inhibitoren können eine Reihe immunvermittelter Nebenwirkungen verursachen, darunter irPruritus (23,0 %), irRash (25,2 %), irFatigue (21,9 %), irHepatitis (15,8 %) sowie irColitis/Gastritis (16,7 %) (Tabelle 3). Wir untersuchten den Zusammenhang zwischen irPruritus und weiteren irAE.

**TABELLE 3:** Prävalenz immunvermittelter Nebenwirkungen und deren Assoziation mit irPruritus. Die statistische Auswertung erfolgte mittels logistischer Regressionsanalyse.

	Alle Patienten (n = 461)	Patienten ohne Juckreiz (n = 355)	Patienten mit Juckreiz (n = 106)	p‐Wert
*Nebenwirkung, n (%)*				
irRash	116 (25,2)	42 (11,8)	74 (69,2)	<0,001
irFatigue	101 (21,9)	67 (18,9)	34 (32,1)	<0,01
irHepatitis	73 (15,8)	53 (14,9)	20 (18,9)	0,6
irColitis/Gastritis	77 (16,7)	58 (16,3)	19 (17,9)	0,7
irHypothyreosis	71 (15,6)	52 (14,6)	20 (18,9)	0,4
irXerostomia	28 (6,1)	23 (6,5)	5 (4,7)	0,5
irPneumonia	27 (5,9)	22 (6,2)	5 (4,7)	0,3
irPancreatitis	24 (5,2)	21 (5,9)	3 (2,8)	0,1
irEdema	12 (2,6)	10 (2,8)	2 (1,9)	0,2
irVitiligo	11 (2,4)	9 (2,5)	2 (1,9)	0,8
irMyasthenia/Myositis	9 (2,0)	7 (2,0)	2 (1,9)	0,7
irNephritis	6 (1,3)	4 (1,1)	2 (1,9)	0,2
irKeratitis sicca	8 (1,7)	8 (2,3)	0,0	0,1
irKeratosis	3 (0,7)	3 (0,8)	0,0	0,1

Von den Patienten mit irPruritus entwickelten 74 (69,2 %) zusätzlich einen irRash und 34 (32,1 %) eine irFatigue. Sowohl irFatigue als auch irRash korrelierten signifikant mit irPruritus (p < 0,001 beziehungsweise p < 0,01). Für keine weiteren irAE ließ sich ein signifikanter Zusammenhang mit irPruritus nachweisen.

## DISKUSSION

Unsere Studie bestätigt die Relevanz von irPruritus während einer Behandlung mit ICI, insbesondere unter Kombinationstherapie. Die Inzidenz von irPruritus unter Monotherapie (Nivolumab 17,6 %, Pembrolizumab 22,1 %) war signifikant niedriger als unter Kombinationstherapie mit Ipilimumab und Nivolumab (31,6 %). Dies bestätigt frühere Untersuchungen in kleineren Kohorten.^16^, ^18^, ^26^ Eine noch höhere Rate von 33,2 % wurde unter Kombinationstherapie bei 104 von 313 Patienten beobachtet.^27^, ^28^

Immunvermittelte Nebenwirkungen als Folge einer Überaktivierung des Immunsystems können Ausdruck einer effektiven Immunstimulation unter Checkpoint‐Inhibitor‐Therapie sein.^29^ Milde irAE mit niedrigem Schweregrad, die ohne Unterbruch der ICI‐Therapie kontrolliert werden können, sind daher potenzielle Indikatoren für ein günstiges Therapieansprechen.^29^, ^30^ In unserer Kohorte waren irPruritus, irXerostomie und irKeratitis sicca mit einem verlängerten Gesamtüberleben assoziiert. Passend dazu war irPruritus überwiegend niedriggradig und führte nur bei einem von 461 Patienten zum Abbruch der Therapie. Weitere Studien zeigten, dass irPruritus Grad 3/4 unter Nivolumab‐Monotherapie bei weniger als 1 % der Patienten auftrat.^31^ Angesichts der hohen Prävalenz von irPruritus Grad 1/2 und dessen Einfluss auf die Lebensqualität der Patienten ist ein besseres Verständnis geeigneter Behandlungsansätze erforderlich. In unserer Kohorte waren TCS zur Behandlung von irPruritus bei 90,9 % der Patienten erfolgreich, während Antihistaminika und topisches Polidocanol eine geringere Wirksamkeit zeigten. Am häufigsten wurden TCS der Klasse III eingesetzt, mit Ansprechen in 93,2 % der Patienten. Die Wahl der Wirkstärke sollte sich am betroffenen Körperareal orientieren, da Faktoren wie Epidermisdicke, Barrierefunktion, behandelte Fläche und die Wirkstoffzusammensetzung den Therapieerfolg beeinflussen.^32^ Angesichts der geringen systemischen Resorption bei kurzfristiger Behandlung mit lokal applizierten TCS können insbesondere Patienten mit lokalisiertem, niedriggradigem irPruritus profitieren. Hier ist das Risiko einer Beeinträchtigung der ICI‐Wirksamkeit vergleichsweise gering. Bei großflächiger Anwendung von TCS ist eine systemische Resorption von über 20 mg Prednisonäquivalent möglich.^32^ Dies unterstreicht den Bedarf einer spezifischen Behandlung des irPruritus bei Patienten, die nicht von TCS, oralen Antihistaminika oder topischem Polidocanol profitieren. Ergänzend zu den in unserer Kohorte eingesetzten pruritusspezifischen Therapien können bei therapierefraktären Fällen GABA‐Analoga in Betracht gezogen werden. Immer häufiger wird Dupilumab (IL4R‐Blockade) verwendet.^21^

Die genaue Ursache des irPruritus unter ICI ist bislang nicht geklärt. In unserer Studie bestanden keine Zusammenhänge zwischen Alter, Geschlecht, medizinischer Vorgeschichte, Melanom‐Stadium oder Lokalisation und der Prävalenz von irPruritus. Ähnliche Ergebnisse sind aus einer kleineren Kohorte von Melanompatienten bekannt.^18^ Auch bei ICI‐Therapie anderer Tumorarten zeigt sich ein vergleichbares Nebenwirkungsprofil mit irPruritus in 10 % bis 14–47 %.^18^, ^33^ Diese Ergebnisse deuten darauf hin, dass irPruritus eine direkte Folge der ICI‐Therapie ist und nicht mit der Ursprungsdiagnose Melanom zusammenhängt.

In unserer Kohorte zeigten Patienten mit irPruritus zum Zeitpunkt des Symptombeginns höhere relative Eosinophilenzahlen als vor Therapiebeginn. Die Rolle der Eosinophilen in der ICI‐Therapie ist bislang unzureichend untersucht. Eosinophile gelten als ein potenzieller prädiktiver Biomarker, der sowohl mit besseren Behandlungsergebnissen als auch mit dem Auftreten von irAE in Verbindung steht.^34^ Eine Hypothese geht von einem direkten Zusammenhang zwischen CTLA‐4 Inhibitoren und irPruritus aus, bedingt durch die verstärkte Immunaktivität in der Haut.^35^, ^36^ Passend dazu fanden sich in Hautbiopsien von acht von neun Patienten mit irPruritus und irRash unter Ipilimumab oberflächliche, perivaskuläre CD4^+^ T‐Zell‐Infiltrate begleitet von Eosinophilen in der Dermis.^35^ Im Einklang mit unseren Ergebnissen wurde zum Zeitpunkt des Auftretens von irPruritus ein Anstieg der Eosinophilenzahl im Blut festgestellt.^6^, ^18^, ^35^ Zukünftige Studien sollten histologische Hautuntersuchungen bei Patienten mit irPruritus in Erwägung ziehen und die Eosinophilenzahlen vor sowie beim Auftreten von irPruritus erfassen. Parallel können die Befunde zwischen Haut und Blut verglichen werden.

Eine prospektive Studie beschrieb irPruritus ohne Begleitexanthem als häufigste kutane irAE Grad 2 oder höher.^37^ Erhöhte IL4‐Spiegel wurden sowohl im Blut als auch in der Haut von Patienten mit irPruritus nachgewiesen.^37^ Zusätzlich war die Zahl der Eosinophilen in juckender Haut ohne Begleitexanthem deutlich höher als in der Kontrollkohorte.^37^ Diese Resultate sind eine starke Grundlage für gezielte Interventionen, wie zum Beispiel die Hemmung von IL4R mit Dupilumab.^21^ In unserer Kohorte mit Patienten mit und ohne irPruritus vor Beginn der Behandlung gleiche Eosinophilenzahlen auf, was darauf hindeutet, dass Eosinophilenzahlen kein prädiktiver Biomarker für irPruritus sind.

Die Analyse aller erhobenen irAE in unserer Patientenkohorte zeigte eine signifikante Assoziation von irPruritus mit irFatigue und irRash (p < 0,01 beziehungsweise p < 0,001). Frühere Studien berichten, dass irFatigue bei 15 %–35 % und irRash bei 15 %–40 % der Patienten mit metastasiertem Melanom unter ICI‐Therapie auftritt.^5^, ^26^, ^28^ Der Zusammenhang zwischen irPruritus und irFatigue lässt sich möglicherweise durch schlafbezogene Beeinträchtigungen infolge des Pruritus erklären, alternativ könnte eine durch ICI induzierte systemische Entzündung eine Rolle spielen.

Als Symptom von irRash wird irPruritus durch die Stimulation polymodaler C‐Nervenfasern in der Haut vermittelt. Er kann direkt durch chemische und mechanische Reize oder indirekt über pruritogene Mediatoren von Mastzellen, T‐Zellen oder Eosinophilen ausgelöst werden. Folglich kann irPruritus sowohl mit als auch ohne Vorliegen eines irRash auftreten.^38^

Diese monozentrische, retrospektive Studie erweitert den aktuellen Wissensstand aufgrund der großen Patientenkohorte. Um die Ergebnisse zu bestätigen und die zugrunde liegenden Mechanismen besser zu verstehen, sollten zukünftig multizentrische, prospektive Studien durchgeführt werden. Unsere Ergebnisse betonen die Bedeutung systematischer klinischer Untersuchungen bei Patienten unter die ICI‐Therapie, um frühe und individuelle Interventionen zu ermöglichen, die sowohl die Fortführung der ICI‐Therapie als auch die Lebensqualität der Patienten sichern.

## DANKSAGUNG

Open access publishing facilitated by Universitat Zurich, as part of the Wiley ‐ Universitat Zurich agreement via the Consortium Of Swiss Academic Libraries.

## INTERESSENKONFLIKT

M.L. reports non–project‐related research support from Roche, Novartis, Bacoba, Scailyte and Oncobit and occasional scientific advisory roles for Roche, Leica, Bacoba, Oncobit and Scailyte. R.D. reports consultancy and/or advisory board activities for Novartis, Merck Sharp & Dohme (MSD), Bristol‐Myers Squibb (BMS), Roche, Amgen, Takeda, Kyowa Kirin, Immunocore, Pierre Fabre, Sun Pharma, Sanofi, Catalym, Second Genome, Regeneron, Alligator, T3 Pharma, MaxiVAX SA, Pfizer, Oncobit and touchIME outside the submitted work. A.R., N.I., P.T., L.K. and B.M.S. declare no conflict of interest.

## Supporting information



Supplementary information
