# Supplementary material for: Inzidenz, Schweregrad und möglicher Pathomechanismus von Immuntherapie‐assoziiertem Pruritus bei Melanompatienten: Incidence, severity and potential pathomechanism of immune‐related pruritus in melanoma patients undergoing immune checkpoint inhibitor therapy
Source: J Dtsch Dermatol Ges. 2026 Nov 10;24(11):1566–74. [Article in German] doi: 10.1111/ddg.70048x (PMC13749580; doi:10.1111/ddg.70048x)
Supplement: Supplementary file 1 [file DDG-24-1566-s001.pdf]

ABBILDUNG S1 im Online-Supplement Gesamtüberleben in Abhängigkeit von immunvermittelten Nebenwirkungen. Die statistische Auswertung erfolgte mittels multivariater Cox-Regression (Proportional-Hazards-Modell) unter Einschluss aller erhobenen immunvermittelten Nebenwirkungen (irAE) als Kovariablen.

Abk.: HR, Hazard Ratio

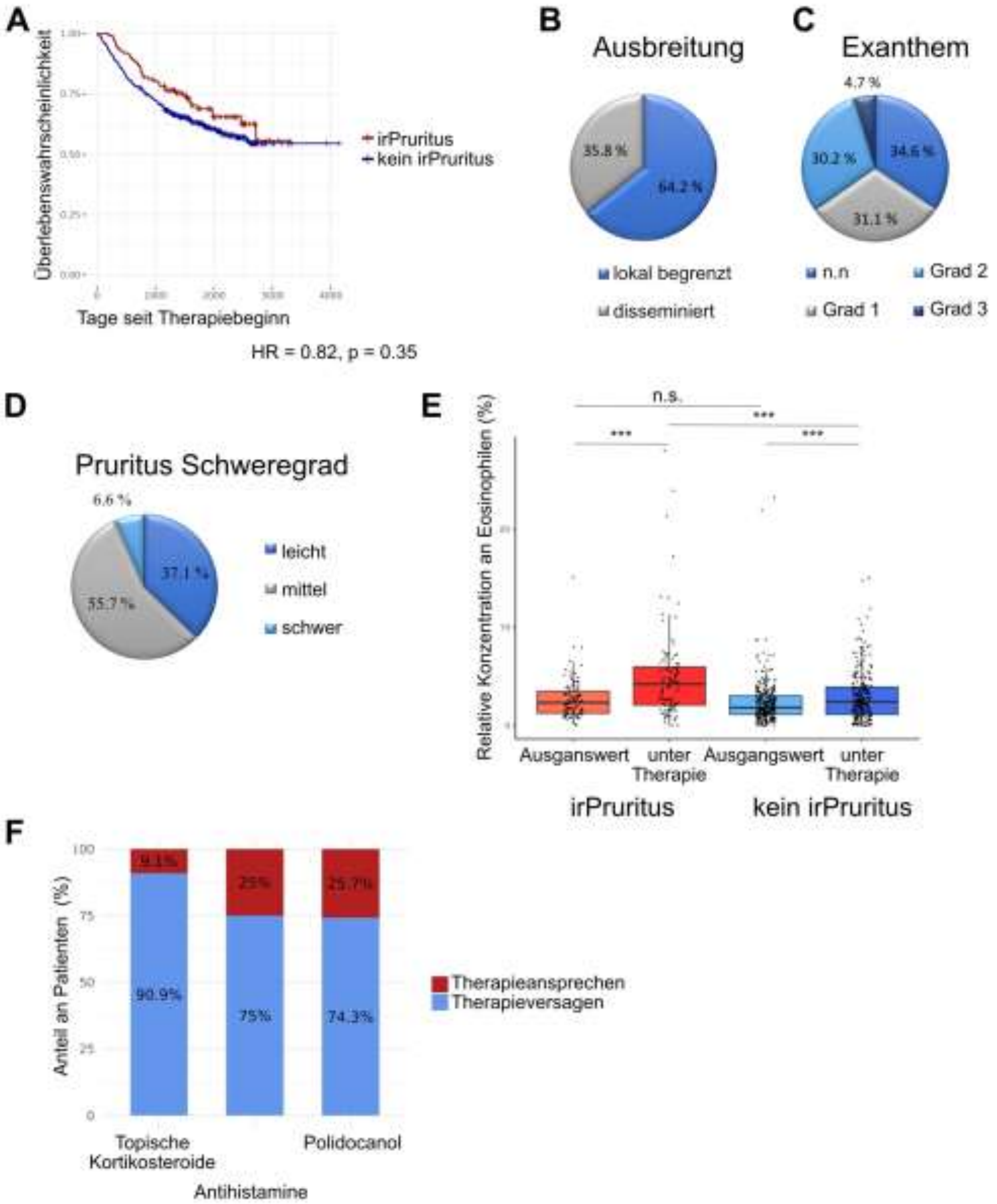

A
Überlebenswahrscheinlichkeit
Tage seit Therapiebeginn
irPruritus
kein irPruritus
HR = 0.82, p = 0.35
B
Ausbreitung
35.8 %
64.2 %
lokal begrenzt
disseminiert
C
Exanthem
4.7 %
30.2 %
34.6 %
31.1 %
n.n
Grad 2
Grad 1
Grad 3
D
Pruritus Schweregrad
6.6 %
37.1 %
55.7 %
leicht
mittel
schwer
E
Relative Konzentration an Eosinophilen (%)
n.s.
***
***
***
Ausganswert
unter Therapie
Ausgangswert
unter Therapie
irPruritus
kein irPruritus
F
Anteil an Patienten (%)
100
75
50
25
0
9.1%
25%
25.7%
90.9%
75%
74.3%
Therapieansprechen
Therapieversagen
Topische Kortikosteroide
Antihistamine
Polidocanol

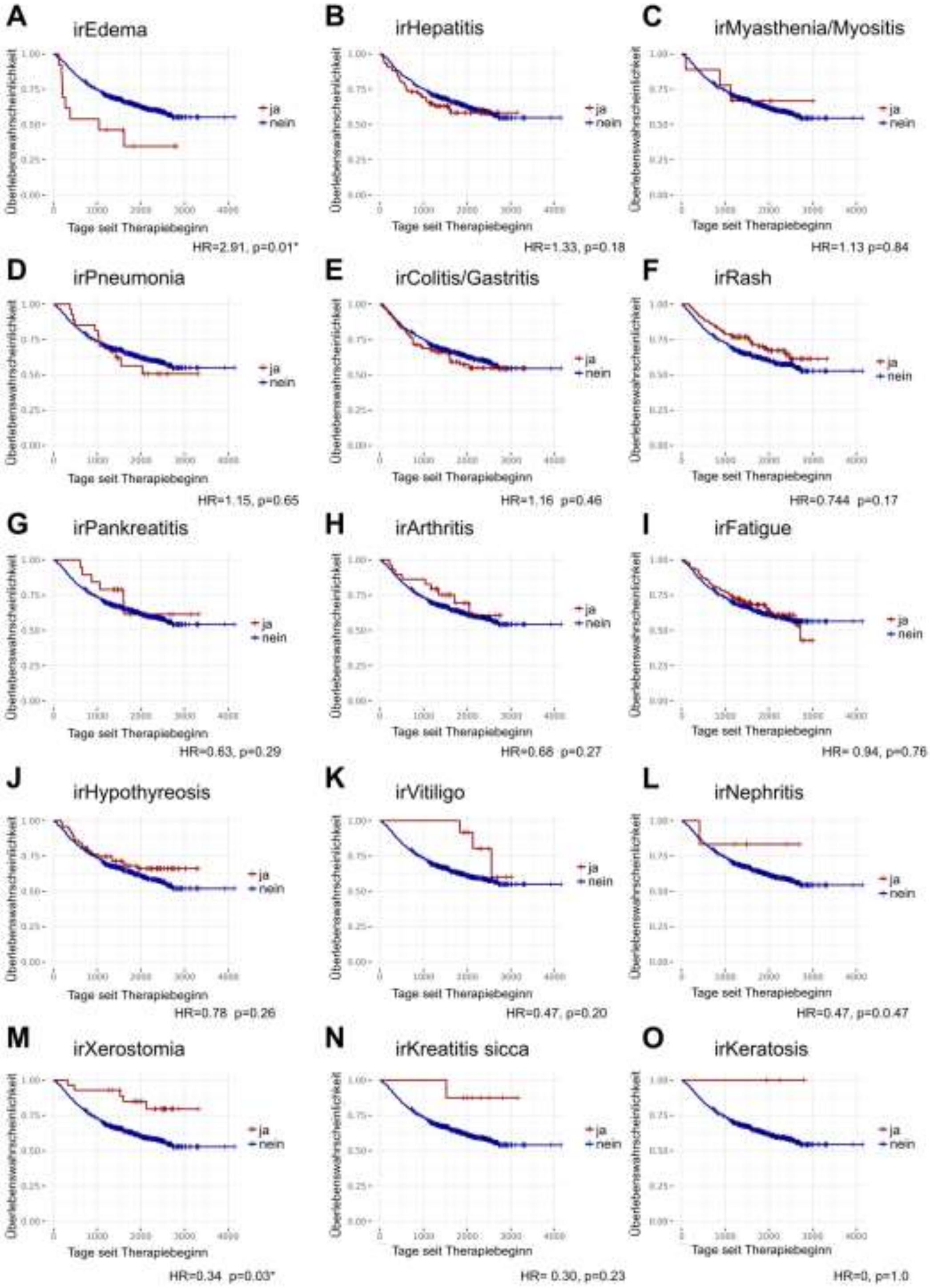

A
irEdema
Überlebenswahrscheinlichkeit
1.00
0.75
0.50
0.25
0.00
0
1000
2000
3000
4000
Tage seit Therapiebeginn
ja
nein
HR=2.91, p=0.01*
B
irHepatitis
HR=1.33, p=0.18
C
irMyasthenia/Myositis
HR=1.13 p=0.84
D
irPneumonia
HR=1.15, p=0.65
E
irColitis/Gastritis
HR=1.16 p=0.46
F
irRash
HR=0.744 p=0.17
G
irPankreatitis
HR=0.63, p=0.29
H
irArthritis
HR=0.68 p=0.27
I
irFatigue
HR= 0.94, p=0.76
J
irHypothyreosis
HR=0.78 p=0.26
K
irVitiligo
HR=0.47, p=0.20
L
irNephritis
HR=0.47, p=0.0.47
M
irXerostomia
HR=0.34 p=0.03*
N
irKreatitis sicca
HR= 0.30, p=0.23
O
irKeratosis
HR=0, p=1.0

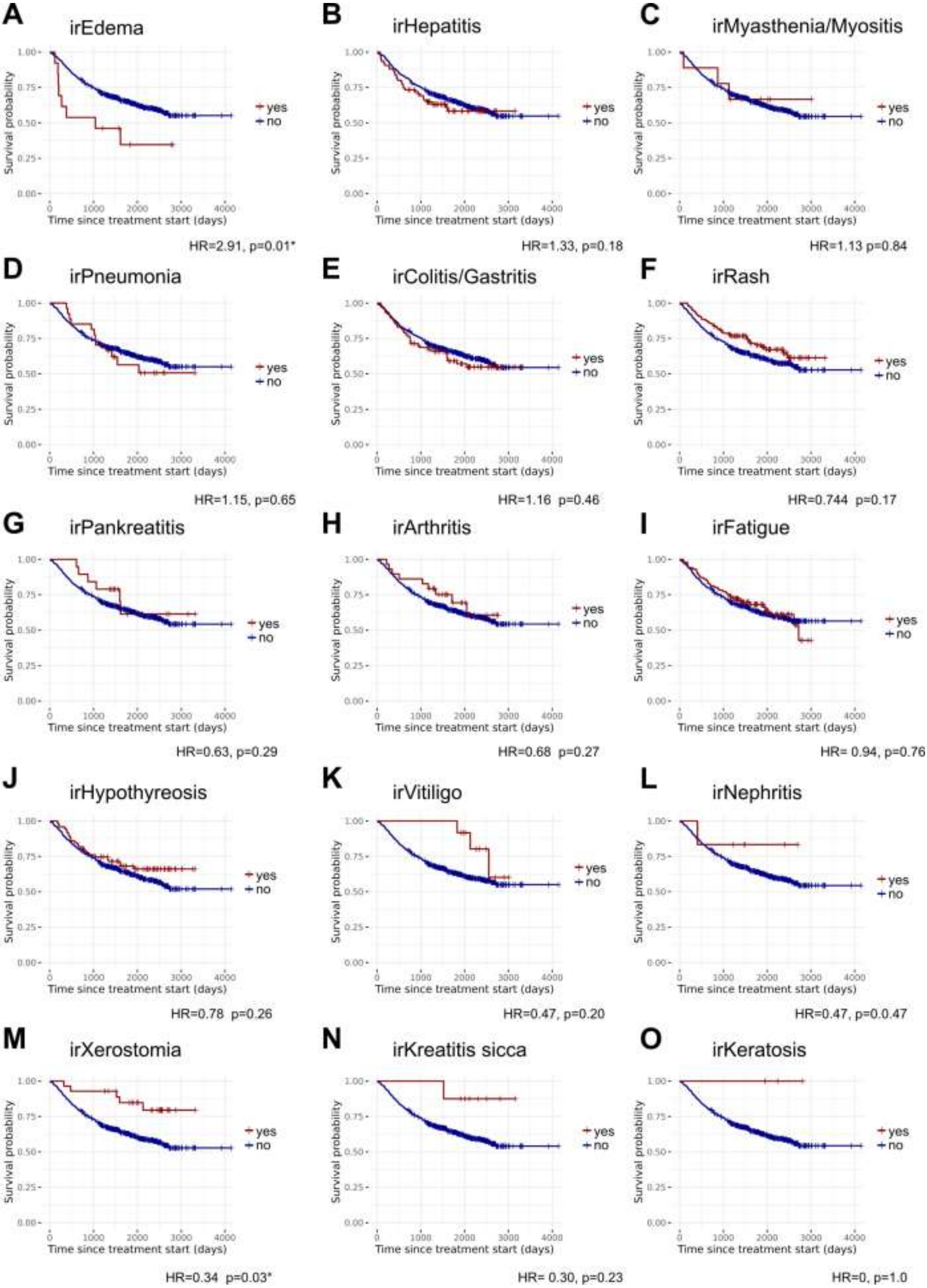
A
irEdema
HR=2.91, p=0.01*
B
irHepatitis
HR=1.33, p=0.18
C
irMyasthenia/Myositis
HR=1.13 p=0.84
D
irPneumonia
HR=1.15, p=0.65
E
irColitis/Gastritis
HR=1.16 p=0.46
F
irRash
HR=0.744 p=0.17
G
irPankreatitis
HR=0.63, p=0.29
H
irArthritis
HR=0.68 p=0.27
I
irFatigue
HR= 0.94, p=0.76
J
irHypothyreosis
HR=0.78 p=0.26
K
irVitiligo
HR=0.47, p=0.20
L
irNephritis
HR=0.47, p=0.0.47
M
irXerostomia
HR=0.34 p=0.03*
N
irKreatitis sicca
HR= 0.30, p=0.23
O
irKeratosis
HR=0, p=1.0
Survival probability
Time since treatment start (days)
yes
no

TABELLE S1 im Online-Supplement Häufigkeit und Behandlungserfolg topischer Kortikosteroide zur Therapie des irPruritus in Abhängigkeit von der Wirkstärke.

Tabelle S1:

	**Therapieansprechen**	**Therapieversagen**
TCS-Klassifizierung n (%)		
I	0	0
II	3 (75,0)	1 (25,0)
III	38 (92,7)	3 (7,3)
IV	9 (90,0)	1 (10,0)

Abk.: TCS, topische Kortikosteroide
